# Cognitive offloading through digital tools and its relationship with critical thinking, task persistence, and learning depth

**DOI:** 10.3389/fpsyg.2026.1781101

**Published:** 2026-03-12

**Authors:** Jian Wang

**Affiliations:** College of Materials and Chemistry & Chemical Engineering, Chengdu University of Technology, Chengdu, China

**Keywords:** cognitive offloading, cognitive self-efficacy, critical thinking, higher education, learning depth, task persistence

## Abstract

**Introduction:**

The increasing reliance on digital tools in university classrooms has raised concerns about cognitive offloading and its potential implications for students' learning processes. Drawing on social cognitive theory, this study investigates how cognitive offloading through digital tools relates to critical thinking, task persistence, and learning depth. It further examines whether cognitive self-efficacy functions as a mediating mechanism linking cognitive offloading to these learning outcomes.

**Methods:**

Data were collected through in-person surveys from undergraduate students enrolled in Chinese universities. Covariance-based structural equation modeling (CB-SEM) was employed to analyze the data. The study first assessed the reliability and validity of the measurement model and then tested the hypothesized mediation framework by comparing competing structural models.

**Results:**

The results show that cognitive offloading is positively associated with cognitive self-efficacy. In turn, cognitive self-efficacy significantly predicts higher levels of critical thinking, task persistence, and learning depth. After accounting for cognitive self-efficacy, the direct effects of cognitive offloading on learning outcomes were reduced. Specifically, cognitive self-efficacy fully mediates the relationship between cognitive offloading and task persistence, and partially mediates the relationships between cognitive offloading and both critical thinking and learning depth.

**Discussion:**

These findings extend the literature by integrating cognitive offloading with self-efficacy theory, highlighting the psychological mechanism through which digital tool use can support meaningful learning outcomes. The study suggests that when students develop stronger cognitive self-efficacy, cognitive offloading through digital tools may facilitate deeper engagement and persistence in learning tasks. Implications are discussed for aligning digital tool use with pedagogical practices that promote critical thinking and deep learning in higher education.

## 1 Introduction

The rapid integration of digital technologies into higher education has fundamentally transformed how students access information, complete academic tasks, and engage in learning. From smartphones and learning management systems to artificial intelligence–based tools, contemporary university classrooms increasingly rely on external digital supports to assist memory, problem solving, and information processing. While these developments offer clear efficiencies, they have also prompted growing scholarly concern regarding whether frequent reliance on digital tools reshapes students' cognitive engagement and learning processes in unintended ways (Li and Lajoie, 2022). Recent research has therefore called for a more nuanced understanding of how technology-mediated learning practices influence higher-order learning outcomes rather than focusing solely on access, usage frequency, or performance gains (Farrell et al., 2024; Oittinen, 2024).

Notably, synthesized evidence from meta-analyses and systematic literature reviews suggests that the effects of digital tool use on higher-order learning outcomes remain fragmented and context-dependent. Meta-analytic findings indicate that although technology-enabled learning environments can support cognitive outcomes, effect sizes vary widely across instructional designs and learning contexts, highlighting the lack of consistent conclusions regarding how and when digital tools enhance learning (Shi et al., 2020; Hu et al., 2025). Complementing these findings, systematic reviews of technology-enhanced and immersive learning environments have noted that existing research tends to emphasize technological features and pedagogical innovations while offering limited insight into the psychological mechanisms that shape learners' engagement, regulation, and deep learning processes (Bizami et al., 2023). Collectively, this body of synthesized evidence underlines the need for theory-driven empirical studies that move beyond descriptive associations and explicitly examine motivational and regulatory pathways through which cognitive offloading in digital environments influences meaningful learning outcomes.

Within this broader discourse, the concept of cognitive offloading has emerged as a central explanatory lens for understanding how individuals delegate cognitive tasks to external tools. Prior studies have primarily examined cognitive offloading from a cognitive and functional perspective, emphasizing its role in reducing memory demands, altering information retention, or redistributing cognitive load (Skulmowski and Xu, 2022; Sun et al., 2025). Although this body of work has provided important foundational insights, it has largely focused on short-term cognitive outcomes or laboratory-based tasks, offering limited evidence on how habitual offloading practices operate in authentic educational settings. As a result, calls have been made for research that situates cognitive offloading within real classroom contexts and examines its implications for meaningful learning outcomes, such as critical thinking (Gerlich, 2025), sustained effort (Turner, 2022), and deep understanding (Skulmowski and Xu, 2022).

At the same time, educational research has consistently emphasized that learning outcomes are not shaped by cognitive processes alone but are deeply intertwined with students' motivational and psychological resources. In particular, students' beliefs about their own cognitive competence have been shown to play a critical role in shaping engagement, persistence, and depth of learning across diverse educational contexts (Hachem et al., 2022). However, despite the growing intersection between digital learning and motivational research, existing studies have rarely examined whether and how technology-related learning behaviors influence these self-beliefs. This omission is notable, as contemporary theories increasingly argue that learning practices and self-perceptions are reciprocally linked, especially in environments characterized by high technological support (Illeris, 2009).

Moreover, prior empirical work has often treated learning outcomes as uniform consequences of technology use, overlooking the possibility that different outcomes may operate through distinct psychological pathways. Research on student learning approaches suggests that outcomes such as critical thinking, task persistence, and learning depth, although related, reflect qualitatively different dimensions of learning that may respond differently to instructional and contextual factors (Almulla and Al-Rahmi, 2023; Andrade et al., 2022; Li et al., 2024). Prior research therefore emphasizes the need for outcome-specific theorizing and the identification of mechanisms that explain why certain learning outcomes are more sensitive to motivational beliefs than others in digitally mediated environments.

Responding to these gaps, the present study integrates cognitive offloading research with social cognitive theory to examine how reliance on digital tools relates to key learning outcomes in university classrooms and to clarify the psychological mechanism underlying these relationships. Rather than assuming that cognitive offloading directly enhances or undermines learning, the study proposes that its effects are transmitted through cognitive self-efficacy, defined as students' beliefs in their ability to think, reason, and solve problems independently. By empirically testing this mediation framework using in-person data from Chinese university students and covariance-based structural equation modeling, the study directly addresses recent calls for theory-driven, context-sensitive investigations of digital learning behaviors. Specifically, this study is among the first to simultaneously examine the mediating roles of cognitive self-efficacy and self-regulated learning control in linking cognitive offloading to multiple higher-order learning outcomes within a single integrative model, thereby extending prior research that has typically focused on isolated outcomes or single mechanisms. In doing so, it advances current understanding of cognitive offloading by embedding it within a broader motivational framework and offers new insights into how digital tool use can be aligned with the development of critical thinking, persistence, and deep learning in higher education.

Subsequently, the study is guided by the following research questions:

RQ1: How does cognitive offloading through digital tools relate to cognitive self-efficacy and self-regulated learning control?

RQ2: How does cognitive offloading through digital tools relate to critical thinking, task persistence, and learning depth?

RQ3: Do cognitive self-efficacy and self-regulated learning control mediate the relationships between cognitive offloading and these learning outcomes?

## 2 Literature review and hypotheses

Contemporary learning theories increasingly recognize that cognition does not operate in isolation from the environment but is shaped by interactions between individuals and external artifacts (Stewart, 2021). From this perspective, digital tools can be understood as extensions of cognitive processes rather than mere aids, a view consistent with distributed cognition and extended mind perspectives. These frameworks argue that thinking is partially offloaded onto external systems, allowing individuals to manage complex tasks more efficiently by reallocating cognitive resources (Skulmowski, 2023). Within educational contexts, this theoretical stance implies that cognitive offloading through digital tools may alter how students engage with learning tasks, not by replacing cognition entirely, but by reshaping the balance between internal effort and external support.

However, extended and distributed cognition theories alone offer limited insight into why similar offloading practices may yield divergent learning outcomes across students. Social cognitive theory provides a complementary lens by emphasizing the central role of self-beliefs in regulating cognition, motivation, and behavior. According to this framework, individuals' beliefs about their cognitive capabilities influence how they approach challenges, persist in the face of difficulty, and engage in deeper processing (Bandura, 1997). Within learning environments, cognitive self-efficacy has been shown to predict higher levels of effort, persistence, and strategic engagement, particularly when tasks demand sustained cognitive involvement (Zimmerman, 2000). This suggests that the consequences of cognitive offloading cannot be fully understood without accounting for how it shapes, and is shaped by, students' beliefs in their own cognitive competence.

Integrating these perspectives offers a more complete theoretical explanation of learning in technology-rich classrooms. While cognitive offloading may reduce immediate cognitive demands, its longer-term educational implications likely depend on whether students interpret offloading as supportive scaffolding or as a substitute for their own thinking. When digital tools are used in ways that reinforce students' confidence in their ability to reason independently, offloading may coexist with, or even strengthen, adaptive learning behaviors. Conversely, when reliance on external tools undermines perceptions of cognitive capability, students may disengage more quickly or exhibit reduced persistence. This logic aligns with research on self-regulated learning, which highlights that effective learning emerges from the interaction of cognitive strategies, motivational beliefs, and contextual supports rather than from any single factor in isolation (Beckmann et al., 2023).

**Figure 1:**
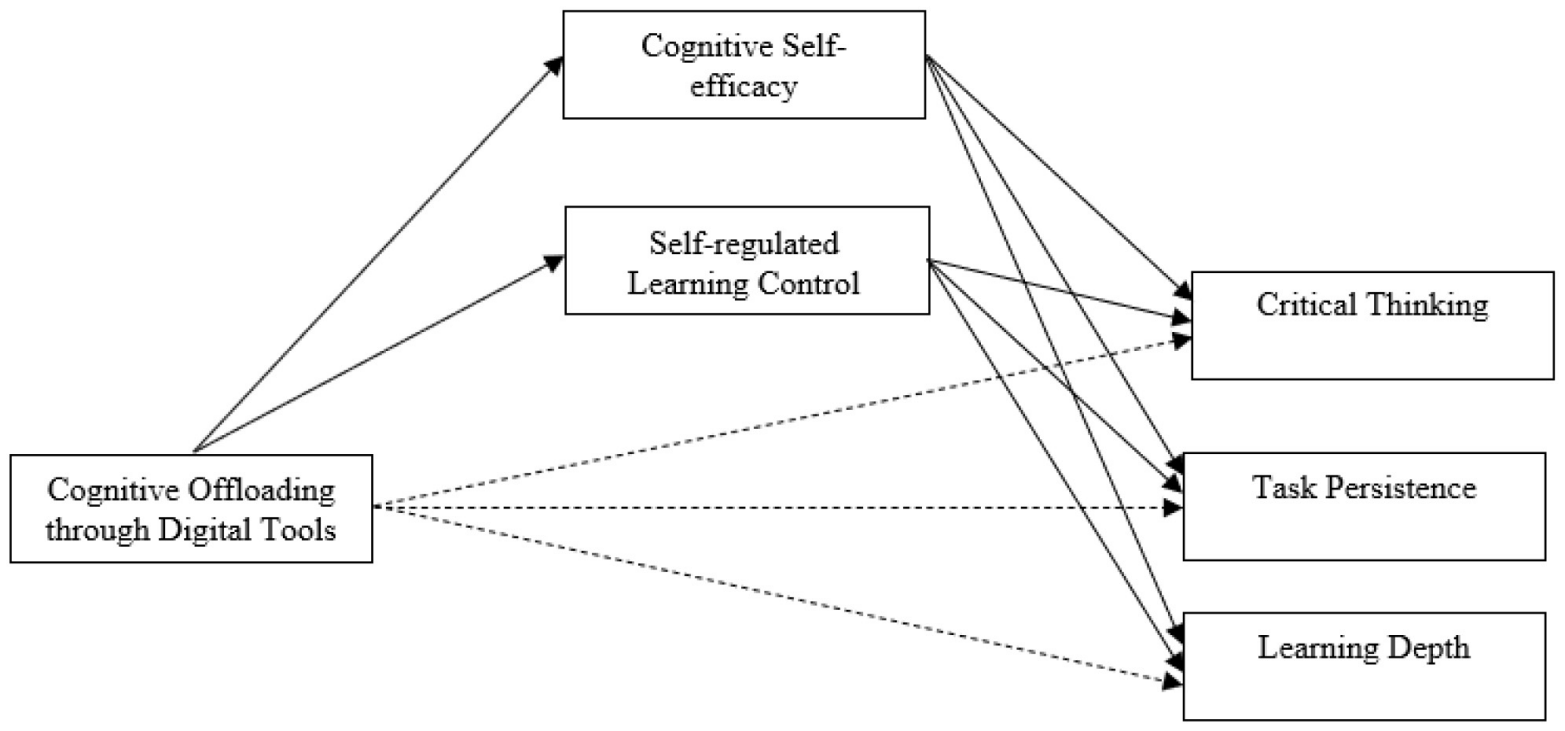
Proposed model.

Finally, theoretical work on learning outcomes highlights the importance of distinguishing among different dimensions of learning when examining technology-related behaviors. Critical thinking, task persistence, and learning depth represent related but conceptually distinct outcomes that reflect analytical reasoning, motivational endurance, and conceptual understanding, respectively (Gaviria Alzate et al., 2024). Theoretical models of learning suggest that motivational beliefs such as self-efficacy may play a particularly central role in sustaining effort over time, whereas higher-order cognitive outcomes may depend on both internal beliefs and external cognitive supports. By situating cognitive offloading within this integrated theoretical framework, the present study provides a coherent basis for examining how digital tool use interacts with cognitive self-efficacy to shape multiple dimensions of learning in higher education (Figure 1).

### 2.1 Linking cognitive offloading through digital tools with cognitive self-efficacy

The increasing reliance on digital tools in academic settings has reshaped how students engage with cognitively demanding tasks, raising important questions about its implications for learners' beliefs in their own cognitive capabilities. From a social cognitive perspective, cognitive self-efficacy develops through repeated experiences of successful task completion, perceived control over problem solving, and confidence in one's ability to manage cognitive challenges (Bandura, 1997). When digital tools are used to support information organization, reduce excessive cognitive load, or scaffold complex reasoning, they may enable students to experience greater cognitive success, thereby reinforcing beliefs in their own cognitive competence. Prior research suggests that technology-assisted learning environments can enhance learners' confidence when tools are perceived as facilitators of understanding rather than substitutes for thinking (Heo et al., 2021; Ma and Lee, 2021; Song and Song, 2023).

At the same time, studies on cognitive offloading indicate that externalizing memory and information-processing demands can free internal cognitive resources, allowing individuals to focus on higher-level reasoning and problem solving (Grinschgl et al., 2021). This redistribution of cognitive effort may foster a sense of cognitive mastery, particularly when learners remain actively engaged in interpreting, evaluating, and applying information retrieved through digital means (Armitage and Gilbert, 2025; Weis and Wiese, 2022). Empirical evidence has shown that students who strategically use digital supports often report higher confidence in managing academic tasks, especially in complex learning environments (Wahn et al., 2023; Zou et al., 2025). Conversely, the absence of such supports may overwhelm learners, undermining their confidence in their own cognitive abilities (Sayaf et al., 2021). Taken together, these perspectives suggest that cognitive offloading through digital tools can positively shape cognitive self-efficacy by enabling learners to experience competence and control in cognitively demanding tasks. Therefore, it is hypothesized that:

H1: Cognitive offloading through digital tools is positively related to cognitive self-efficacy.

### 2.2 Linking cognitive offloading through digital tools with self-regulated learning

Beyond its influence on self-beliefs, cognitive offloading may also shape how students regulate their learning processes. Self-regulated learning theory emphasizes learners' active role in planning, monitoring, and adjusting their cognitive and behavioral strategies to achieve academic goals (Zimmerman, 2000). Digital tools that support note-taking, task scheduling, progress tracking, and information retrieval can serve as external regulatory resources, assisting learners in organizing learning activities and monitoring task progress. Prior research suggests that technology-enhanced learning environments can strengthen self-regulatory behaviors by providing timely feedback, structure, and cues for strategic adjustment (Bains et al., 2022; Sui et al., 2024; Urbina et al., 2021).

From this perspective, cognitive offloading does not merely reduce cognitive effort but may function as a regulatory aid that complements learners' internal control processes. By delegating routine or memory-intensive components of tasks to digital tools, students may devote greater attention to goal setting, strategy selection, and performance monitoring, which are core elements of self-regulated learning (Wei, 2023). Empirical studies have shown that learners who effectively integrate digital tools into their study routines demonstrate higher levels of metacognitive awareness and learning regulation, particularly in complex or information-rich contexts (Chang et al., 2023; Chiu, 2023; Lee and Hwang, 2022). However, such benefits depend on the purposeful and strategic use of technology rather than passive reliance. Drawing on these insights, cognitive offloading through digital tools is expected to support learners' regulatory capacity by enhancing planning and monitoring processes. Accordingly, it is hypothesized that:

H2: Cognitive offloading through digital tools is positively related to self-regulated learning.

### 2.3 Cognitive self-efficacy as a mediating variable

In digitally mediated learning environments, the influence of cognitive offloading on students' learning outcomes is unlikely to be direct or uniform; rather, it is shaped by learners' internal cognitive and motivational resources. Theoretical perspectives grounded in social cognitive theory emphasize that beliefs about one's cognitive capability play a central role in determining how individuals approach complex tasks, persist through difficulty, and engage in deeper cognitive processing (Bandura, 1997). Within this framework, cognitive self-efficacy functions as a key psychological mechanism that translates learning experiences into observable outcomes, particularly in contexts where external supports are readily available.

Research on cognitive offloading suggests that delegating memory and information-processing demands to digital tools can alter how learners allocate cognitive effort, often freeing resources for higher-order reasoning and problem solving (Josué et al., 2023; Sudarma et al., 2022). When such offloading is experienced as supportive rather than substitutive, learners may interpret their successful task performance as evidence of their own cognitive competence, thereby strengthening cognitive self-efficacy. Empirical studies in technology-enhanced and blended learning contexts support this view, showing that students who use digital tools strategically report greater confidence in handling complex academic tasks and exhibit more adaptive learning behaviors (Ma and Lee, 2021; Warren et al., 2021; Windasari et al., 2024).

Cognitive self-efficacy, in turn, has been consistently linked to a range of learning outcomes. Students with stronger efficacy beliefs are more likely to engage in analytical reasoning, critically evaluate information, and integrate new knowledge with existing understanding, which are core components of critical thinking and deep learning (AL-Qadri et al., 2024; Shamdas, 2023). Moreover, cognitive self-efficacy is a robust predictor of motivational endurance, as confident learners are more willing to persist when tasks are challenging or cognitively demanding (Chou et al., 2024). Studies focusing on digital and online learning environments further corroborate that self-efficacy plays a decisive role in determining whether access to technological supports leads to sustained engagement and meaningful learning rather than superficial task completion (Liu and Duan, 2022; Rehman et al., 2024; Saleh and AlAli, 2022).

Taken together, these theoretical arguments and empirical findings suggest that cognitive offloading through digital tools influences learning outcomes primarily by shaping students' beliefs in their own cognitive capability. Rather than exerting strong direct effects, cognitive offloading is expected to operate indirectly, enhancing critical thinking, task persistence, and learning depth insofar as it strengthens cognitive self-efficacy. Accordingly, the following hypotheses are proposed:

H3a: Cognitive self-efficacy mediates the relationship between cognitive offloading through digital tools and critical thinking.

H3b: Cognitive self-efficacy mediates the relationship between cognitive offloading through digital tools and task persistence.

H3c: Cognitive self-efficacy mediates the relationship between cognitive offloading through digital tools and learning depth.

### 2.4 Self-regulated learning control as a mediating variable

In technology-rich learning environments, the effects of cognitive offloading on learning outcomes are increasingly understood as operating through learners' capacity to manage and regulate their own learning processes (Luo and Zhou, 2024). Self-regulated learning theory emphasizes that effective learning depends on learners' ability to plan, monitor, and adjust their cognitive and behavioral strategies in response to task demands (Geng and Su, 2025). From this perspective, learning outcomes such as critical thinking, task persistence, and learning depth are not merely products of information access or cognitive support, but of learners' regulatory engagement with tasks over time.

Digital tools that enable cognitive offloading—such as note-taking applications, search engines, task planners, and learning management systems—may function as external regulatory aids rather than simple cognitive substitutes. By delegating routine memory or organizational demands to digital systems, learners can redirect attention toward goal setting, progress monitoring, and strategic decision making, which are core components of self-regulated learning control (Lobos et al., 2024). Empirical research suggests that students who integrate digital tools into their study routines in a deliberate manner exhibit stronger planning behaviors, greater metacognitive awareness, and more adaptive strategy use (Alvarez et al., 2022; Khalil et al., 2024; Mejeh et al., 2024).

Importantly, self-regulated learning control has been consistently linked to higher-order learning outcomes across educational contexts. Learners who actively regulate their learning are more likely to engage in critical analysis, persist through difficulty, and adopt deep learning approaches that emphasize conceptual understanding over surface memorization (Alhalafawy and Zaki, 2022; Mohanraj, 2024). In digitally mediated environments, these regulatory processes become even more salient, as the abundance of information and tools increases the need for effective monitoring and control (Mejeh et al., 2024). In addition, studies in online and blended learning contexts demonstrate that the benefits of technology use for learning are contingent on learners' self-regulatory capacity rather than on tool availability alone (Eggers et al., 2021; Lobos et al., 2024; Stebner et al., 2022).

Taken together, this body of research suggests that cognitive offloading through digital tools may enhance learning outcomes by strengthening learners' self-regulated learning control. Rather than directly improving critical thinking, persistence, or learning depth, cognitive offloading is expected to operate indirectly by supporting learners' ability to organize, monitor, and strategically manage their learning activities. Accordingly, self-regulated learning control is proposed as a key mediating mechanism linking cognitive offloading to multiple dimensions of learning. Based on these theoretical arguments and prior empirical evidence, the following hypotheses are proposed:

H4a: Self-regulated learning control mediates the relationship between cognitive offloading through digital tools and critical thinking.

H4b: Self-regulated learning control mediates the relationship between cognitive offloading through digital tools and task persistence.

H4c: Self-regulated learning control mediates the relationship between cognitive offloading through digital tools and learning depth.

## 3 Methodology

### 3.1 Participants and procedures

The study was conducted in mainland China and targeted undergraduate students enrolled in regular university programs. Data were collected through an in-person survey administration to ensure higher response accuracy, reduce common method concerns (Podsakoff et al., 2003). Prior to data collection, permission was obtained from relevant academic units, and instructors facilitated access to classrooms during scheduled sessions. Trained research assistants visited classrooms and briefed students about the voluntary nature of participation, anonymity of responses, and academic purpose of the study. Only students who provided informed consent were included.

The participating institutions included public universities representing different tiers of the Chinese higher education system, including comprehensive and applied universities. Students were recruited from general undergraduate courses across social sciences, humanities, sciences, engineering, and management disciplines. Although the courses were not exclusively technology-intensive, digital tools such as learning management systems, mobile devices, online academic platforms, and course-related applications were routinely integrated into instructional activities, reflecting typical technology use in contemporary Chinese university classrooms.

A probability-based sampling approach was adopted to enhance representativeness. Specifically, stratified random sampling was used, where universities were first grouped by institutional type and academic discipline, and intact classes were then randomly selected within each stratum. This approach was suitable given the structured classroom environment and allowed adequate coverage of students from different academic backgrounds while maintaining feasibility for in-person administration.

In total, 600 students were approached across multiple universities. Of these, 512 questionnaires were returned, yielding a high response rate attributable to in-class administration. After screening for incomplete responses, patterned answering, and excessive missing data, 468 questionnaires were retained for final analysis. The final sample size was considered adequate for covariance-based structural equation modeling using AMOS. This decision was guided by common SEM recommendations that emphasize an appropriate ratio of observations to estimated parameters, model complexity, and the need for stable covariance matrices. Given the number of latent constructs and observed indicators in the proposed model, a sample exceeding 400 respondents was deemed sufficient to ensure reliable parameter estimation and acceptable statistical power (Hair et al., 2021).

To statistically assess potential common method bias, Harman's single-factor test was conducted as a diagnostic procedure. The results showed that the first unrotated factor accounted for 32.6% of the total variance, which is below the recommended threshold of 40%, suggesting that common method variance was unlikely to pose a serious threat to the validity of the findings (Podsakoff et al., 2003).

Regarding demographic characteristics, the sample reflected a balanced composition of university students. Approximately 53% of respondents were female and 47% were male. In terms of age, about 62% were between 18 and 20 years, 31% were between 21 and 23 years, and the remaining 7% were aged 24 years or above. With respect to academic year, first-year students accounted for roughly 28% of the sample, second-year students 34%, third-year students 27%, and final-year students 11%. Participants represented diverse academic disciplines, with around 36% enrolled in social sciences and management-related programs, 29% in science and engineering fields, 21% in humanities and arts, and 14% in other applied disciplines.

## 4 Measures

All study variables were measured using previously validated instruments, with minor contextual wording adjustments to ensure suitability for the Chinese university classroom context. All items were translated into Chinese following a standard translation and back-translation procedure to preserve semantic equivalence (Brislin, 1980). Responses were recorded on a five-point Likert scale ranging from 1 (strongly disagree) to 5 (strongly agree), unless stated otherwise (Appendix A).

### 4.1 Cognitive offloading through digital tools

Cognitive offloading through digital tools was assessed by adapting items that capture the extent to which individuals rely on external digital devices to store information, support memory, and assist thinking processes. The scale reflects habitual tendencies to delegate cognitive tasks to technologies such as smartphones, note-taking applications, and online search tools. Items were adapted from prior work on cognitive offloading and technology-assisted cognition, which conceptualizes offloading as a functional extension of human cognitive processes rather than simple technology use (Risko and Gilbert, 2016; Sparrow et al., 2011). Sample items focused on reliance on digital tools for remembering information and completing academic tasks.

### 4.2 Cognitive self-efficacy

Cognitive self-efficacy was measured using a well-established self-efficacy framework grounded in social cognitive theory. The scale captured students' confidence in their ability to think independently, solve complex problems, and understand difficult academic material without excessive reliance on external aids. Items were adapted from generalized and academic self-efficacy measures that have been widely used in educational and learning research (Bandura, 1997; Pintrich and De Groot, 1990). Higher scores indicated stronger beliefs in one's own cognitive capabilities.

### 4.3 Self-regulated learning control

Self-regulated learning control was measured by assessing students' perceived ability to plan, monitor, and regulate their learning activities. The scale emphasized metacognitive control, goal setting, and self-monitoring during learning tasks. Items were adapted from established self-regulated learning instruments that conceptualize learning control as an active and intentional process guiding academic engagement (Zimmerman, 2000; Zimmerman and Schunk, 2011). Sample items reflected students' tendencies to evaluate their understanding and adjust learning strategies when facing difficulties.

### 4.4 Critical thinking

Critical thinking was assessed using items that reflect analytical reasoning, evaluation of arguments, and reflective judgment in academic contexts. The scale focused on students' ability to question assumptions, integrate evidence, and draw reasoned conclusions. Items were adapted from widely cited critical thinking measures used in higher education research (Facione, 1990; Halpern, 1998), ensuring alignment with classroom-based cognitive demands.

### 4.5 Task persistence

Task persistence was measured by capturing students' sustained effort and perseverance when facing challenging or time-consuming academic tasks. The scale emphasized consistency of effort and resistance to disengagement, drawing on prior conceptualizations of persistence as a core motivational and behavioral outcome in learning settings (Duckworth et al., 2007; Credé et al., 2017). Items assessed students' willingness to continue working despite difficulty or frustration.

### 4.6 Learning depth

Learning depth was assessed using items that reflect deep learning approaches, including conceptual understanding, integration of ideas, and meaningful knowledge construction. The scale distinguished deep learning from surface-level memorization and focused on understanding underlying principles and applying knowledge across contexts. Items were adapted from established deep learning and learning approaches scales commonly used in higher education research (Biggs, 1987; Entwistle and Ramsden, 2015). Higher scores indicated a greater tendency toward deep and meaningful learning.

## 5 Control

Several control variables were included to account for potential confounding effects on students' learning outcomes. Gender, age, academic year, and academic discipline were controlled because prior research suggests that these demographic factors may influence technology use patterns, self-regulatory behaviors, and cognitive engagement in university settings. Additionally, prior academic performance was controlled to partial out baseline differences in learning ability that could affect critical thinking, task persistence, and learning depth independent of cognitive offloading and identity-related mechanisms.

## 6 Analysis

The proposed model includes multiple latent constructs and indirect pathways, requiring an analytical approach capable of simultaneously estimating measurement and structural relationships while explicitly accounting for measurement error. Structural equation modeling is well suited for such theory-driven models, as it enables the testing of complex causal structures involving mediation within a single, coherent framework (Kline, 2016). A covariance-based approach using AMOS is particularly appropriate when the objective is theory confirmation and the assessment of overall model fit rather than prediction (Hair et al., 2021). Moreover, CB-SEM allows for rigorous evaluation of factor structure, construct validity, and structural relationships based on covariance matrices, which aligns with the reflective nature of the constructs in this study (Byrne, 2013). Given the adequate sample size relative to model complexity, the use of AMOS provides sufficient statistical power and stable parameter estimation for examining the hypothesized relationships in higher education research contexts (Boomsma and Hoogland, 2001; Wolf et al., 2013).

## 7 Results

The results of the measurement model are presented in Table 1. The six-factor confirmatory factor analysis demonstrated an acceptable fit to the data, as indicated by a chi-square to degrees of freedom ratio of 2.07, which is below the recommended upper limit of 3.0. Incremental fit indices further supported model adequacy, with comparative fit index values of 0.948 for the measurement model and 0.945 for the hypothesized mediation model, and Tucker–Lewis index values of 0.941 and 0.938, respectively, exceeding the commonly accepted threshold of 0.90. Absolute fit indices were also satisfactory, with root mean square error of approximation values of 0.048 for the measurement model and 0.047 for the mediation model, both accompanied by narrow 90% confidence intervals, and standardized root mean square residual values of 0.051 and 0.053, respectively, remaining well below the 0.08 criterion. In contrast, the direct-effects-only model and the misspecified alternative model exhibited weaker fit, as reflected in lower CFI values (0.889 and 0.861) and elevated RMSEA values (0.072 and 0.081), indicating that these models provided a less adequate representation of the data. Overall, these results support the adequacy of the measurement model and the superiority of the hypothesized mediation structure, consistent with established SEM fit guidelines (Hu and Bentler, 1999; Kline, 2016).

**Table 1:** Model fit indices for competing measurement and structural models.

**Model**	**Description**	***χ^2^***	**df**	***χ^2^*/df**	**CFI**	**TLI**	**RMSEA (90% CI)**	**SRMR**
M1	Measurement model (six-factor CFA)	1,274.20	614	2.07	0.948	0.941	0.048 (0.044, 0.052)	0.051
M2	Hypothesized mediation model	1,319.60	638	2.07	0.945	0.938	0.047 (0.043, 0.051)	0.053
M3	Structural model (direct effects only)	1,586.30	640	2.48	0.889	0.873	0.072 (0.068, 0.076)	0.089
M4	Alternative misspecified model	1,712.80	640	2.67	0.861	0.845	0.081 (0.077, 0.085)	0.104

Table 2 reports the reliability and convergent validity statistics for all constructs. Cronbach's alpha values ranged from 0.85 to 0.90, and composite reliability values ranged from 0.88 to 0.92, exceeding the recommended minimum of 0.70 and indicating strong internal consistency across all measures Hair et al., 2019. Convergent validity was supported, as average variance extracted values ranged from 0.54 to 0.60, surpassing the 0.50 threshold and demonstrating that each construct accounted for a substantial proportion of variance in its indicators (Fornell and Larcker, 1981). In addition, maximum shared variance values ranged from 0.31 to 0.38 and were consistently lower than the corresponding average variance extracted values, providing further evidence that each construct shared more variance with its own indicators than with other constructs in the model.

**Table 2:** Construct reliability and convergent validity.

**Construct**	**α**	**CR**	**AVE**	**MSV**
Cognitive offloading	0.86	0.88	0.55	0.32
Cognitive self-efficacy	0.89	0.91	0.58	0.34
Self-regulated learning control	0.87	0.89	0.56	0.36
Critical thinking	0.90	0.92	0.60	0.38
Task persistence	0.85	0.88	0.54	0.31
Learning depth	0.88	0.90	0.57	0.35

Discriminant validity was further assessed using the Fornell-Larcker criterion, as shown in Table 3. The square roots of the average variance extracted, which ranged from 0.73 to 0.77, were greater than the corresponding inter-construct correlations, which ranged from 0.44 to 0.63. For example, the square root of the average variance extracted for cognitive self-efficacy was 0.76, exceeding its highest correlation with another construct (0.62 with critical thinking), and the square root of the average variance extracted for learning depth was 0.75, exceeding its correlation with critical thinking (0.63). This pattern satisfies the Fornell-Larcker criterion and confirms that the constructs are empirically distinct despite their theoretically related nature (Fornell and Larcker, 1981).

**Table 3:** Discriminant validity: Fornell-Larcker.

**Construct**	**1**	**2**	**3**	**4**	**5**	**6**
1. Cognitive offloading	**0.74**					
2. Cognitive self-efficacy	0.56	**0.76**				
3. Self-regulated learning control	0.52	0.60	**0.75**			
4. Critical thinking	0.48	0.62	0.58	**0.77**		
5. Task persistence	0.44	0.57	0.55	0.59	**0.73**	
6. Learning depth	0.46	0.59	0.57	0.63	0.61	**0.75**

Diagonal elements (in bold) represent the square roots of the average variance extracted (AVE). Off-diagonal elements represent inter-construct correlations.

The structural path estimates reported in Table 4 provide direct evidence for the hypothesized mediation relationships. In the direct-effects-only model, cognitive offloading exhibited significant positive associations with critical thinking (β = 0.25, *p* = 0.000), task persistence (β = 0.19, *p* = 0.002), and learning depth (β = 0.23, *p* = 0.000), indicating that reliance on digital tools was initially related to all three learning outcomes. However, in the mediation model, cognitive offloading showed a significant positive effect on cognitive self-efficacy (β = 0.31, *p* = 0.000), and cognitive self-efficacy, in turn, demonstrated strong associations with critical thinking (β = 0.41, *p* = 0.000), task persistence (β = 0.36, *p* = 0.000), and learning depth (β = 0.33, *p* = 0.000). After accounting for cognitive self-efficacy, the direct effect of cognitive offloading on task persistence became non-significant (β = 0.08, *p* = 0.159), while its effects on critical thinking (β = 0.12, *p* = 0.043) and learning depth (β = 0.10, *p* = 0.082) were substantially reduced. This attenuation pattern is consistent with mediation logic in structural equation modeling, whereby the inclusion of a theoretically meaningful mediator explains variance previously attributed to direct effects (Baron and Kenny, 1986; Kline, 2016).

**Table 4:** Structural path estimates across competing models.

**Structural path**	**Direct-effects model (M3) Std. β**	**C.R**.	***p***	**Mediation model (M2) Std. β**	**C.R**.	***p***
Cognitive Offloading → Cognitive self-efficacy	—	—	—	0.31	4.54	0.000
Cognitive Offloading → Self-regulated learning control	—	—	—	0.27	4.01	0.000
Cognitive self-efficacy → Critical thinking	—	—	—	0.41	6.78	0.000
Cognitive self-efficacy → Task persistence	—	—	—	0.36	6.02	0.000
Cognitive self-efficacy → Learning depth	—	—	—	0.33	5.48	0.000
Self-regulated learning control → Critical thinking	—	—	—	0.29	4.96	0.000
Self-regulated learning control → Task persistence	—	—	—	0.34	5.71	0.000
Self-regulated learning control → Learning depth	—	—	—	0.31	5.22	0.000
Cognitive offloading → Critical thinking	0.25	4.11	0.000	0.09	1.68	0.093
Cognitive offloading → Task persistence	0.19	3.09	0.002	0.06	1.21	0.226
Cognitive offloading → Learning depth	0.23	3.78	0.000	0.08	1.49	0.136

Std. β, standardized regression weight; C.R., critical ratio. M3 represents the direct-effects-only structural model, whereas M2 represents the hypothesized mediation model. p-values are two-tailed and based on maximum likelihood estimation in AMOS.

In addition, the mediation model incorporated self-regulated learning control as a parallel mediating mechanism. Cognitive offloading exhibited a significant positive association with self-regulated learning control (β = 0.27, *p* = 0.000), indicating that greater reliance on digital tools was linked to stronger regulatory control over learning activities. Self-regulated learning control, in turn, demonstrated significant positive relationships with critical thinking (β = 0.29, *p* = 0.000), task persistence (β = 0.34, *p* = 0.000), and learning depth (β = 0.31, *p* = 0.000). When both mediators were included simultaneously, the direct paths from cognitive offloading to all three learning outcomes became non-significant, providing additional support for the presence of indirect effects operating through self-regulated learning control alongside cognitive self-efficacy. This pattern is consistent with contemporary mediation approaches emphasizing the role of motivational and regulatory processes in explaining technology–learning relationships (Hayes, 2018; Kline, 2016).

The mediation effects were formally tested using bias-corrected bootstrap procedures, as reported in Table 5. The indirect effect of cognitive offloading on critical thinking via cognitive self-efficacy was significant [β = 0.13, 95% CI (0.06, 0.21), *p* = 0.001], as was the indirect effect on task persistence [β = 0.11, 95% CI (0.05, 0.19), *p* = 0.003] and learning depth [β = 0.10, 95% CI (0.04, 0.18), *p* = 0.006]. In all cases, the confidence intervals did not include zero, providing robust evidence of mediation (Hayes, 2018). Taken together, these findings indicate partial mediation for critical thinking and learning depth, where both direct and indirect effects were present, and full mediation for task persistence, where the direct effect became non-significant once cognitive self-efficacy was included in the model.

**Table 5:** Bootstrap results for indirect (mediation) effects.

**Indirect path**	**Indirect effect (Std. β)**	**Boot SE**	**95% CI (LL)**	**95% CI (UL)**	***p***
Cognitive offloading → Cognitive self-efficacy → Critical thinking	0.13	0.04	0.06	0.21	0.001
Cognitive offloading → Cognitive self-efficacy → Task persistence	0.11	0.04	0.05	0.19	0.003
Cognitive offloading → Cognitive self-efficacy → Learning depth	0.10	0.04	0.04	0.18	0.006
Cognitive offloading → Self-regulated learning control → Critical thinking	0.08	0.03	0.03	0.15	0.004
Cognitive offloading → Self-regulated learning control → Task persistence	0.09	0.03	0.04	0.16	0.002
Cognitive offloading → Self-regulated learning control → Learning depth	0.08	0.03	0.03	0.14	0.005

Indirect effects were estimated using bias-corrected bootstrap procedures with 5,000 resamples.

CI, confidence interval; LL, lower limit; UL, upper limit.

Parallel bootstrap analyses further revealed significant indirect effects through self-regulated learning control. Specifically, the indirect effect of cognitive offloading on critical thinking via self-regulated learning control was significant [β = 0.08, 95% CI (0.03, 0.15), *p* = 0.004], as were the indirect effects on task persistence [β = 0.09, 95% CI (0.04, 0.16), *p* = 0.002] and learning depth [β = 0.08, 95% CI (0.03, 0.14), *p* = 0.005]. The absence of zero within all corresponding confidence intervals provides additional support for mediation. Consistent with contemporary mediation frameworks, these findings indicate that self-regulated learning control operates as an additional explanatory pathway through which cognitive offloading influences learning outcomes, complementing the mediating role of cognitive self-efficacy (Hayes, 2018; Kline, 2016).

## 8 Discussion

The findings of the present study provide a nuanced understanding of how cognitive offloading through digital tools operates within university classrooms by clarifying both its direct and indirect relationships with key learning outcomes, summarized in Table 6. Consistent with H1, cognitive offloading was positively associated with cognitive self-efficacy, suggesting that reliance on digital tools does not necessarily weaken students' confidence in their cognitive abilities. Instead, when digital tools are used as supportive resources, they may enable students to manage cognitive demands more effectively, thereby reinforcing their beliefs about their capacity to think, reason, and solve problems. This finding aligns with prior work showing that well-integrated technological support can foster learners' sense of competence rather than undermine it (Bandura, 1997; Panigrahi et al., 2022).

**Table 6:** Summary of hypotheses testing results.

**Hypothesis**	**Path**	**Result**	**Evidence**
H1	Cognitive offloading → Cognitive self-efficacy	Supported	Significant positive path (β = 0.31, *p* = 0.000; Table 4)
H2	Cognitive offloading → Self-regulated learning control	Supported	Significant positive path (β = 0.27, *p* = 0.000; Table 4)
H3a	Cognitive offloading → Cognitive self-efficacy → Critical thinking	Supported	Significant indirect effect [β = 0.13, 95% CI (0.06, 0.21); Table 5]
H3b	Cognitive offloading → Cognitive self-efficacy → Task persistence	Supported	Significant indirect effect [β = 0.11, 95% CI (0.05, 0.19); Table 5]
H3c	Cognitive offloading → Cognitive self-efficacy → Learning depth	Supported	Significant indirect effect [β = 0.10, 95% CI (0.04, 0.18); Table 5]
H4a	Cognitive offloading → Self-regulated learning control → Critical thinking	Supported	Significant indirect effect [β = 0.08, 95% CI (0.03, 0.15); Table 5]
H4b	Cognitive offloading → Self-regulated learning control → Task persistence	Supported	Significant indirect effect [β = 0.09, 95% CI (0.04, 0.16); Table 5]
H4c	Cognitive offloading → Self-regulated learning control → Learning depth	Supported	Significant indirect effect [β = 0.08, 95% CI (0.03, 0.14); Table 5]

Hypothesis support is determined based on the significance of standardized path coefficients and bias-corrected bootstrap confidence intervals that do not include zero.

In support of H2, cognitive offloading was also positively related to self-regulated learning control. This result indicates that offloading cognitive demands to digital tools may free attentional and cognitive resources that students can redirect toward planning, monitoring, and regulating their learning activities. Rather than promoting passivity, digital offloading appears to coexist with greater regulatory control over learning processes, a finding that resonates with research emphasizing the role of external supports in facilitating self-regulation when learners remain actively engaged (Zimmerman, 2008; Andrade et al., 2022).

The mediation analyses further clarify how these relationships translate into learning outcomes. Supporting H3a–H3c, cognitive self-efficacy significantly mediated the relationships between cognitive offloading and critical thinking, task persistence, and learning depth. The results showed that the indirect effects through cognitive self-efficacy were significant across all three outcomes, indicating that students' confidence in their cognitive abilities constitutes a central pathway through which cognitive offloading influences learning. Importantly, the direct effect of cognitive offloading on task persistence became non-significant once cognitive self-efficacy was included in the model, suggesting full mediation. This pattern implies that persistence in academic tasks is particularly dependent on students' beliefs in their own cognitive competence, consistent with prior evidence linking self-efficacy to sustained effort and perseverance (Warren et al., 2021; Prifti, 2022).

In contrast, the relationships between cognitive offloading and both critical thinking and learning depths were only partially mediated by cognitive self-efficacy. Although the direct effects were substantially reduced, they did not disappear entirely. This suggests that higher-order cognitive outcomes may be shaped by both internal beliefs and the structural affordances provided by digital tools, such as access to diverse information sources and opportunities for comparison and reflection. Previous studies have similarly noted that critical thinking and deep learning can be directly influenced by how learners interact with digital information environments, beyond motivational factors alone (Skulmowski and Xu, 2022; Almulla and Al-Rahmi, 2023).

Beyond cognitive self-efficacy, the inclusion of self-regulated learning control as a parallel mediator provides additional insight into the mechanisms underlying cognitive offloading effects. Supporting H4a–H4c, significant indirect effects were observed from cognitive offloading to all three learning outcomes via self-regulated learning control. These findings indicate that cognitive offloading also operates by enhancing students' ability to regulate their learning processes, including goal setting, strategy use, and self-monitoring. This pathway was evident across critical thinking, task persistence, and learning depth, underscoring the importance of regulatory processes in digitally mediated learning environments (Zimmerman, 2008; Li et al., 2024).

Taken together, the results suggest that cognitive offloading through digital tools does not exert a uniform or simplistic influence on learning outcomes. Instead, its effects are transmitted through complementary psychological pathways involving both motivational beliefs and regulatory control. While task persistence appears to be primarily driven by students' confidence in their cognitive abilities, critical thinking and learning depth reflect a more complex interplay between self-beliefs, self-regulation, and direct engagement with digital resources. This differentiated pattern helps explain why prior research has reported mixed findings regarding the educational consequences of digital tool use and highlights the importance of examining outcome-specific mechanisms rather than assuming homogeneous effects (Martin et al., 2022; Yu et al., 2022).

### 8.1 Theoretical implications

The findings of this study extend prior research on cognitive offloading by moving beyond its predominantly cognitive and functional conceptualizations to incorporate a psychologically grounded explanatory mechanism. Earlier work has largely examined cognitive offloading as an externalization of memory and information processing, emphasizing its role in reducing cognitive load or altering memory performance (Jiang and Yu, 2025; Lodge et al., 2023; Skulmowski and Xu, 2022; Xin and Zhang, 2024; Xu et al., 2023). While these studies have provided foundational insights into how digital tools reshape cognitive processes, they have paid limited attention to how habitual offloading practices influence learners' self-perceptions as competent thinkers. By demonstrating that cognitive self-efficacy mediates the relationship between cognitive offloading and learning outcomes, the present study advances the literature by integrating cognitive offloading research with social cognitive theory, thereby shifting theoretical attention from external cognitive aids to learners' internal belief systems (Bandura, 1997).

The study also contributes to self-efficacy and learning theory by clarifying how cognitive self-efficacy is not merely an antecedent of academic outcomes but is itself shaped by students' engagement with digital technologies. Prior studies have consistently linked self-efficacy to critical thinking, persistence, and deep learning, typically treating self-efficacy as a relatively stable personal resource that predicts academic engagement and performance (Panigrahi et al., 2022; Prifti, 2022; Warren et al., 2021). The present findings extend this stream of research by empirically showing that everyday learning behaviors, such as cognitive offloading through digital tools, can actively influence students' confidence in their own cognitive capabilities. This perspective aligns with and expands social cognitive theory by illustrating a reciprocal pathway in which learning practices and self-beliefs mutually shape one another in digitally mediated environments (Bandura, 1997).

Furthermore, the differentiated mediation patterns observed across learning outcomes refine existing theoretical assumptions regarding digital learning and student engagement. Prior research has often examined learning outcomes in aggregate terms or has assumed uniform effects of technology use across cognitive and motivational domains (Kang and Kim, 2021; Martin et al., 2022; Yu et al., 2022). In contrast, the present study shows that task persistence is fully explained by cognitive self-efficacy, whereas critical thinking and learning depth retain partial direct associations with cognitive offloading. This nuanced pattern suggests that persistence-related outcomes are more strongly dependent on learners' self-beliefs, while higher-order cognitive outcomes are influenced by both internal beliefs and external cognitive supports. By revealing these outcome-specific pathways, the study encourages more fine-grained theorizing in future research and cautions against overly generalized models of digital learning behavior.

In addition, the assessment of self-regulated learning control as a parallel mediating mechanism extends existing theoretical accounts by linking cognitive offloading to self-regulated learning theory. Whereas, prior cognitive offloading research has rarely considered learners' regulatory capacities, the present findings suggest that offloading cognitive demands may support learners' ability to plan, monitor, and regulate their learning processes. This integration bridges cognitive offloading theory with self-regulated learning perspectives, highlighting that digital tool use can simultaneously shape motivational beliefs and regulatory control mechanisms in technology-rich learning environments (Zimmerman, 2008).

Taken together, the study aligns with earlier theoretical frameworks on cognition, motivation, and learning, while extending them by introducing cognitive self-efficacy as a central mechanism linking cognitive offloading to educational outcomes. In doing so, it offers a more integrated theoretical account of learning in contemporary classrooms, where digital tools are ubiquitous and students' beliefs about their own cognitive competence play a decisive role in determining whether technology use enhances or undermines meaningful learning.

### 8.2 Practical implications

The findings of this study yield several practical implications for educators, instructional designers, and higher education institutions seeking to optimize learning in technology-rich classrooms. First, the results suggest that cognitive offloading through digital tools should not be treated simply as a risk to students' thinking skills or as an unqualified instructional aid; rather, its effectiveness depends critically on whether it strengthens or weakens students' cognitive self-efficacy. In practice, this implies that instructors should deliberately design learning activities that encourage students to use digital tools as supports for thinking rather than as substitutes for it. For example, educators can require students to explain, justify, or reflect on solutions generated with the help of digital tools, thereby reinforcing students' confidence in their own cognitive abilities while still leveraging technological affordances.

Second, the strong mediating role of cognitive self-efficacy highlights the importance of instructional strategies that explicitly cultivate students' beliefs in their own cognitive competence. Universities and instructors can integrate formative feedback, mastery-oriented assessments, and scaffolded challenges that gradually increase task complexity, helping students' experience successful independent thinking even in digitally mediated tasks. By reinforcing students' sense of cognitive capability, these practices may enhance task persistence and sustained effort, particularly in demanding academic contexts.

Finally, the differentiated effects observed across learning outcomes suggest that a one-size-fits-all approach to technology integration is unlikely to be effective. While digital tools may directly support certain aspects of higher-order thinking, students' persistence appears to depend more strongly on their internal beliefs than on technology use *per se*. Consequently, institutional policies and professional development programs should emphasize pedagogical training that helps instructors balance digital tool use with strategies aimed at fostering learner autonomy, self-belief, and reflective thinking. By aligning technology integration with psychological principles of motivation and learning, higher education institutions can ensure that cognitive offloading practices contribute to meaningful, sustained learning rather than superficial engagement.

### 8.3 Limitations and research directions

Despite its contributions, the study has several limitations that suggest directions for future research. First, the cross-sectional design restricts causal inferences, as the observed relationships capture associations at a single point in time; longitudinal or experimental designs would be valuable for examining how cognitive offloading and cognitive self-efficacy co-evolve over time. Second, the reliance on self-reported measures may introduce common method bias and subjective evaluation, particularly in assessing technology use and cognitive beliefs; future studies could incorporate behavioral indicators, learning analytics, or performance-based assessments to strengthen measurement robustness. Third, the sample was drawn from university students in China, which may limit the generalizability of the findings to other cultural or educational contexts; cross-cultural replications could help determine whether the identified mechanisms operate similarly in different learning environments. In addition, future research could adopt experimental designs that explicitly manipulate different forms of digital offloading, such as generative artificial intelligence tools, automated problem solvers, or basic digital note-taking, to examine whether distinct offloading modalities differentially influence self-efficacy, self-regulated learning, and learning outcomes. Finally, although the study focused on cognitive self-efficacy as a central mechanism, other psychological processes such as metacognitive awareness, epistemic beliefs, or motivation orientations may also play important roles and warrant examination in more complex or sequential mediation models.

## 9 Conclusion

In conclusion, this study provides empirical evidence that cognitive offloading through digital tools influences university students' learning outcomes primarily through its impact on cognitive self-efficacy. By integrating cognitive offloading research with social cognitive theory, the findings demonstrate that reliance on digital tools does not automatically undermine or enhance learning; instead, its effects depend on whether students maintain confidence in their own cognitive capabilities. The results further reveal that cognitive self-efficacy fully explains the link between cognitive offloading and task persistence and partially explains its relationship with critical thinking and learning depth, underscoring the central role of self-beliefs in digitally mediated learning environments. In this sense, the study is among the first to empirically demonstrate that cognitive offloading through digital tools shapes university students' learning outcomes through distinct psychological pathways involving cognitive self-efficacy and self-regulated learning control. Together, these insights contribute to a more nuanced understanding of how technology use, psychological resources, and learning outcomes intersect in contemporary higher education and highlight the importance of aligning digital tool use with pedagogical practices that strengthen students' sense of cognitive competence.

## Data Availability

The original contributions presented in the study are included in the article/supplementary material, further inquiries can be directed to the corresponding author.

## Appendix A.

Measurement items. All constructs were measured using multi-item scales. Responses to all items were recorded on a five-point Likert scale ranging from 1 = strongly disagree to 5 = strongly agree.

**Cognitive offloading through digital tools**

1. I rely on digital tools to remember important academic information.
2. I use digital devices to store information instead of keeping it in my memory.
3. When solving academic tasks, I depend on digital tools to handle complex information.
4. I prefer using digital tools to reduce the mental effort required for learning tasks.
5. I often delegate thinking or remembering tasks to digital applications.
6. Digital tools help me manage cognitive demands during my studies.

**Cognitive self-efficacy**

1. I am a creative person when dealing with academic tasks.
2. I am confident in my ability to think through complex academic problems.
3. I am good at generating effective solutions to learning-related challenges.
4. Compared to my classmates, my ideas are often of high quality.
5. I believe I can develop effective solutions for almost any academic problem.
6. I feel confident when I need to use my thinking skills to solve difficult tasks.
7. I am capable of handling learning tasks that require sustained mental effort.

**Self-regulated learning control**

1. I plan my learning activities carefully before starting academic tasks.
2. I monitor my understanding while studying.
3. I adjust my learning strategies when I realize something is not working.
4. I set clear goals for my learning tasks.
5. I evaluate my learning progress regularly.
6. I am able to control my learning behavior even when tasks are difficult.

**Critical thinking**

1. I carefully evaluate the credibility of information before accepting it.
2. I analyze issues from multiple perspectives before forming conclusions.
3. I question assumptions underlying arguments or explanations.
4. I reflect on the reasoning behind my conclusions to ensure they are logical.
5. I use evidence to support my academic judgments.
6. I think critically about the information I encounter in my coursework.

**Task persistence**

1. I continue working on academic tasks even when they become difficult.
2. I persist with challenging learning activities until they are completed.
3. I do not give up easily when faced with demanding coursework.
4. I put sustained effort into completing complex academic tasks.
5. I keep trying even when I encounter obstacles in my learning.

**Learning depth**

1. I try to understand the underlying meaning of what I learn rather than just memorizing facts.
2. I relate new knowledge to concepts I have learned previously.
3. I seek to integrate ideas from different sources when studying.
4. I focus on gaining a deep understanding of course material.
5. I reflect on how different concepts are connected.
6. I aim to learn in ways that allow me to apply knowledge to new situations.

## References

[B1] Alhalafawy W. Zaki M. T. (2022). How has gamification within digital platforms affected self-regulated learning skills during the COVID-19 pandemic? Mixed-methods research. Int. J. Emerg. Technol. Learn. 17, 123–151. doi: 10.3991/ijet.v17i06.28885

[B2] Almulla M. A. Al-Rahmi W. M. (2023). Integrated social cognitive theory with learning input factors: the effects of problem-solving skills and critical thinking skills on learning performance sustainability. Sustainability 15:3978. doi: 10.3390/su15053978

[B3] AL-Qadri A. H. Mouas S. Saraa N. Boudouaia A. (2024). Measuring academic self-efficacy and learning outcomes: the mediating role of university English students' academic commitment. Asian Pac. J. Second Foreign Lang. Educ. 9:35. doi: 10.1186/s40862-024-00253-5

[B4] Alvarez R. P. Jivet I. Perez-Sanagustin M. Scheffel M. Verbert K. (2022). Tools designed to support self-regulated learning in online learning environments: a systematic review. IEEE Trans. Learn. Technol. 15, 508–522. doi: 10.1109/TLT.2022.3193271

[B5] Andrade M. S. Miller R. M. McArthur D. Ogden M. (2022). The impact of learning on student persistence in higher education. J. Coll. Stud. Retent. Res. Theory Pract. 24, 316–336. doi: 10.1177/1521025120915576

[B6] Armitage K. L. Gilbert S. J. (2025). The nature and development of cognitive offloading in children. Child Dev. Perspect. 19, 108–115. doi: 10.1111/cdep.12532

[B7] Bains M. Kaliski D. Z. Goei K. A. (2022). Effect of self-regulated learning and technology-enhanced activities on anatomy learning, engagement, and course outcomes in a problem-based learning program. Adv. Physiol. Educ. 46, 219–227. doi: 10.1152/advan.00039.2021 35113679

[B8] Bandura A. (1997). Self-Efficacy: The Exercise of Control. New York, NY: Freeman.

[B9] Baron R. M. Kenny D. A. (1986). The moderator–mediator variable distinction in social psychological research: conceptual, strategic, and statistical considerations. J. Pers. Soc. Psychol. 51, 1173–1182. doi: 10.1037/0022-3514.51.6.1173 3806354

[B10] Beckmann J. Beckmann-Waldenmayer D. Wolf S. A. (2023). “Self-regulation in competitive sports,” in Sport and Exercise Psychology: Theory and Application, eds. J. Schüler, M. Wegner, H. Plessner, and R. C. Eklund (Cham: Springer International Publishing), 491–512.

[B11] Biggs J. B. (1987). Student Approaches to Learning and Studying. Melbourne: Australian Council for Educational Research.

[B12] Bizami N. A. Tasir Z. Kew S. N. (2023). Innovative pedagogical principles and technological tools capabilities for immersive blended learning: a systematic literature review. Educ. Inf. Technol. 28, 1373–1425. doi: 10.1007/s10639-022-11243-w 35919874 PMC9334534

[B13] Boomsma A. Hoogland J. J. (2001). “The robustness of LISREL modeling revisited,” in Structural Equation Modeling: Present and Future, eds. R. Cudeck, S. du Toit, and D. Sörbom (Chicago, IL: Scientific Software International), 139–168.

[B14] Brislin R. W. (1980). “Translation and content analysis of oral and written material,” in Handbook of Cross-Cultural Psychology: Methodology, eds. H. C. Triandis and J. W. Berry (Boston, MA: Allyn and Bacon), 389–444.

[B15] Byrne B. M. (2013). Structural Equation Modeling with AMOS: Basic Concepts, Applications, and Programming, 3rd Edn. New York, NY: Routledge.

[B16] Chang D. H. Lin M. P. C. Hajian S. Wang Q. Q. (2023). Educational design principles of using AI chatbot that supports self-regulated learning in education: goal setting, feedback, and personalization. Sustainability 15:12921. doi: 10.3390/su151712921

[B17] Chiu T. K. (2023). Student engagement in K-12 online learning amid COVID-19: a qualitative approach from a self-determination theory perspective. Interact. Learn. Environ. 31, 3326–3339. doi: 10.1080/10494820.2021.1926289

[B18] Chou S. W. Hsieh M. C. Pan H. C. (2024). Understanding the impact of self-regulation on perceived learning outcomes based on social cognitive theory. Behav. Inf. Technol. 43, 1129–1148. doi: 10.1080/0144929X.2023.2198048

[B19] Credé M. Tynan M. C. Harms P. D. (2017). Much ado about grit: a meta-analytic synthesis of the grit literature. J. Pers. Soc. Psychol. 113, 492–511. doi: 10.1037/pspp0000102 27845531

[B20] Duckworth A. L. Peterson C. Matthews M. D. Kelly D. R. (2007). Grit: perseverance and passion for long-term goals. J. Pers. Soc. Psychol. 92, 1087–1101. doi: 10.1037/0022-3514.92.6.1087 17547490

[B21] Eggers J. H. Oostdam R. Voogt J. (2021). Self-regulation strategies in blended learning environments in higher education: a systematic review. Australas. J. Educ. Technol. 37, 175–192. doi: 10.14742/ajet.6453

[B22] Entwistle N. Ramsden P. (2015). Understanding Student Learning. London, UK: Croom Helm.

[B23] Facione P. A. (1990). Critical Thinking: A Statement of Expert Consensus for Purposes of Educational Assessment and Instruction. Millbrae, CA: California Academic Press.

[B24] Farrell T. Alani H. Mikroyannidis A. (2024). Mediating learning with learning analytics technology: guidelines for practice. Teach. High. Educ. 29, 1500–1520. doi: 10.1080/13562517.2022.2067745

[B25] Fornell C. Larcker D. F. (1981). Evaluating structural equation models with unobservable variables and measurement error. J. Market. Res. 18, 39–50. doi: 10.1177/002224378101800104

[B26] Gaviria Alzate S. J. Valencia-Sánchez W. G. Arias Arias E. A. (2024). The critical thinking approach to tactical development in team sports: a review of the work of Jean Francis Gréhaigne. Phys. Educ. Sport Ped. 1–26. doi: 10.1080/17408989.2024.2432312

[B27] Geng X. Su Y. S. (2025). The effects of different metacognitive patterns on students' self-regulated learning in blended learning. Comput. Educ. 227:105211. doi: 10.1016/j.compedu.2024.105211

[B28] Gerlich M. (2025). AI tools in society: Impacts on cognitive offloading and the future of critical thinking. Societies 15:6. doi: 10.3390/soc15010006

[B29] Grinschgl S. Papenmeier F. Meyerhoff H. S. (2021). Consequences of cognitive offloading: boosting performance but diminishing memory. Q. J. Exp. Psychol. 74, 1477–1496. doi: 10.1177/17470218211008060 33752519 PMC8358584

[B30] Hachem M. Gorgun G. Chu M. W. Bulut O. (2022). Social and emotional variables as predictors of students' perceived cognitive competence and academic performance. Canad. J. Sch. Psychol. 37, 362–384. doi: 10.1177/08295735221118474

[B31] Hair J. F. Astrachan C. B. Moisescu O. I. Radomir L. Sarstedt M. Vaithilingam S. . (2021). Executing and interpreting applications of PLS-SEM: updates for family business researchers. J. Fam. Bus. Strat. 12:100392. doi: 10.1016/j.jfbs.2020.100392

[B32] Hair J. F. Risher J. J. Sarstedt M. Ringle C. M. (2019). When to use and how to report the results of PLS-SEM. Eur. Bus. Rev. 31, 2–24. doi: 10.1108/EBR-11-2018-0203

[B33] Halpern D. F. (1998). Teaching for transfer: learning and teaching for understanding. Am. Psychol. 53, 449–455. 9572008 10.1037//0003-066x.53.4.449

[B34] Hayes A. F. (2018). Introduction to Mediation, Moderation, and Conditional Process Analysis: A Regression-Based Approach, 2nd Edn. New York, NY: Guilford Press.

[B35] Heo H. Bonk C. J. Doo M. Y. (2021). Enhancing learning engagement during COVID-19 pandemic: self-efficacy in time management, technology use, and online learning environments. J. Comput. Assist. Learn. 37, 1640–1652. doi: 10.1111/jcal.12603

[B36] Hu L. Zhang W. Lin P. (2025). Can the utilization of technology-enhanced learning spaces lead to improved learning outcomes? A meta-analysis based on 39 experimental and quasi-experimental studies. Interact. Learn. Environ. 33, 3052–3072. doi: 10.1080/10494820.2024.2436943

[B37] Hu L. T. Bentler P. M. (1999). Cutoff criteria for fit indexes in covariance structure analysis: conventional criteria versus new alternatives. Struct. Equ. Model. 6, 1–55. doi: 10.1080/10705519909540118

[B38] Illeris K. (2009). Contemporary Theories of Learning, Vol. 2. London: Routledge.

[B39] Jiang X. Yu Z. (2025). Exploring the impact of learner-and peer-help seeking strategies on cognitive load reduction and self-regulated learning skills enhancement. Interact. Learn. Environ. 33, 4062–4081. doi: 10.1080/10494820.2025.2457352

[B40] Josué A. Bedoya-Flores M. C. Mosquera-Quiñonez E. F. Mesías-Simisterra Á. E. Bautista-Sánchez J. V. (2023). Educational platforms: digital tools for the teaching-learning process in education. Ibero Am. J. Educ. Soc. Res. 3, 259–263. doi: 10.56183/iberoeds.v3i1.626

[B41] Kang H. Y. Kim H. R. (2021). Impact of blended learning on learning outcomes in the public healthcare education course: a review of flipped classroom with team-based learning. BMC Med. Educ. 21:78. doi: 10.1186/s12909-021-02508-y 33509176 PMC7845047

[B42] Khalil M. Wong J. Wasson B. Paas F. (2024). Adaptive support for self-regulated learning in digital learning environments. Br. J. Educ. Technol. 55, 1281–1289. doi: 10.1111/bjet.13479

[B43] Kline R. B. (2016). Principles and Practice of Structural Equation Modeling, 4th Edn. New York, NY: Guilford Press.

[B44] Lee H. Hwang Y. (2022). Technology-enhanced education through VR-making and metaverse-linking to foster teacher readiness and sustainable learning. Sustainability 14:4786. doi: 10.3390/su14084786

[B45] Li S. Lajoie S. P. (2022). Cognitive engagement in self-regulated learning: an integrative model. Eur. J. Psychol. Educ. 37, 833-852. doi: 10.1007/s10212-021-00565-x

[B46] Li T. Ji Y. Zhan Z. (2024). Expert or machine? Comparing the effect of pairing student teacher with in-service teacher and ChatGPT on their critical thinking, learning performance, and cognitive load in an integrated-STEM course. Asia Pac. J. Educ. 44, 45–60. doi: 10.1080/02188791.2024.2305163

[B47] Liu L. Duan Z. (2022). Influences of environmental perception on individual cognitive engagement in online learning: the mediating effect of self-efficacy. Int. J. Emerg. Technol. Learn. 17, 66–78. doi: 10.3991/ijet.v17i04.29221

[B48] Lobos K. Cobo-Rendón R. Bruna Jofré D. Santana J. (2024). New challenges for higher education: self-regulated learning in blended learning contexts. Front. Educ. 9:1457367. doi: 10.3389/feduc.2024.1457367

[B49] Lodge J. M. Yang S. Furze L. Dawson P. (2023). It's not like a calculator, so what is the relationship between learners and generative artificial intelligence?. Learn. Res. Pract. 9, 117–124. doi: 10.1080/23735082.2023.2261106

[B50] Luo R. Z. Zhou Y. L. (2024). The effectiveness of self-regulated learning strategies in higher education blended learning: a five years systematic review. J. Comput. Assist. Learn. 40, 3005–3029. doi: 10.1111/jcal.13052

[B51] Ma L. Lee C. S. (2021). Evaluating the effectiveness of blended learning using the ARCS model. J. Comput. Assist. Learn. 37, 1397–1408. doi: 10.1111/jcal.12579

[B52] Martin F. Wu T. Wan L. Xie K. (2022). A meta-analysis on the community of inquiry presences and learning outcomes in online and blended learning environments. Online Learn. 26, 325–359. doi: 10.24059/olj.v26i1.2604

[B53] Mejeh M. Sarbach L. Hascher T. (2024). Effects of adaptive feedback through a digital tool–a mixed-methods study on the course of self-regulated learning. Educ. Inf. Technol. 29, 1–43. doi: 10.1007/s10639-024-12510-8 PMC11511727 39464525

[B54] Mohanraj S. G. (2024). “Defining the potentials of self-regulated learning through digital environments: a theoretical perspective,” in Transforming Education for the 21st Century-Innovative Teaching Approaches (Chennai: Orange Books Publication), 110.

[B55] Oittinen T. (2024). Highlighting as a referential and collaborative practice in multiparty video-mediated learning activities. Classroom Discourse 15, 180–202. doi: 10.1080/19463014.2023.2259020

[B56] Panigrahi R. Srivastava P. R. Panigrahi P. K. Dwivedi Y. K. (2022). Role of internet self-efficacy and interactions on blended learning effectiveness. J. Comput. Inf. Syst. 62, 1239–1252. doi: 10.1080/08874417.2021.2004565

[B57] Pintrich P. R. De Groot E. V. (1990). Motivational and self-regulated learning components of classroom academic performance. J. Educ. Psychol. 82, 33–40. doi: 10.1037/0022-0663.82.1.33

[B58] Podsakoff P. M. MacKenzie S. B. Lee J. Y. Podsakoff N. P. (2003). Common method biases in behavioral research: a critical review of the literature and recommended remedies. J. Appl. Psychol. 88:879. doi: 10.1037/0021-9010.88.5.879 14516251

[B59] Prifti R. (2022). Self–efficacy and student satisfaction in the context of blended learning courses. Open Learn. 37, 111–125. doi: 10.1080/02680513.2020.1755642

[B60] Rehman S. Addas A. Rehman E. Khan M. N. Shahiman M. A. Rahman M. A. . (2024). Leveraging digital skills to reduce cognitive strain: implications for academic self-efficacy in medical education. Acta Psychol. 251:104602. doi: 10.1016/j.actpsy.2024.104602 39541913

[B61] Risko E. F. Gilbert S. J. (2016). Cognitive offloading. Trends Cogn. Sci. 20, 676–688. doi: 10.1016/j.tics.2016.07.002 27542527

[B62] Saleh S. AlAli R. (2022). Digital learning tools (institutional-open) and their relationship to educational self-effectiveness and achievement in online learning environments. Przestrz. Społecz. 22, 226–256.

[B63] Sayaf A. M. Alamri M. M. Alqahtani M. A. Al-Rahmi W. M. (2021). Information and communications technology used in higher education: an empirical study on digital learning as sustainability. Sustainability 13:7074. doi: 10.3390/su13137074

[B64] Shamdas G. (2023). The relationship between academic self-efficacy and cognitive learning outcomes of high school students in biology subjects through problem-based learning model. J. Penelit. Pendidik. IPA 9, 5466–5473. doi: 10.29303/jppipa.v9i7.3018

[B65] Shi Y. Yang H. MacLeod J. Zhang J. Yang H. H. (2020). College students' cognitive learning outcomes in technology-enabled active learning environments: a meta-analysis of the empirical literature. J. Educ. Comput. Res. 58, 791–817. doi: 10.1177/0735633119881477

[B66] Skulmowski A. (2023). The cognitive architecture of digital externalization. Educ. Psychol. Rev. 35:101. doi: 10.1007/s10648-023-09818-1

[B67] Skulmowski A. Xu K. M. (2022). Understanding cognitive load in digital and online learning: a new perspective on extraneous cognitive load. Educ. Psychol. Rev. 34, 171–196. doi: 10.1007/s10648-021-09624-7

[B68] Skulmowski A. Xu K. M. (2022). Understanding cognitive load in digital and online learning: a new perspective on extraneous cognitive load. Educ. Psychol. Rev. 34, 171–196. doi: 10.1007/s10648-021-09624-7

[B69] Song C. Song Y. (2023). Enhancing academic writing skills and motivation: assessing the efficacy of ChatGPT in AI-assisted language learning for EFL students. Front. Psychol. 14:1260843. doi: 10.3389/fpsyg.2023.1260843 38162975 PMC10754989

[B70] Sparrow B. Liu J. Wegner D. M. (2011). Google effects on memory: cognitive consequences of having information at our fingertips. Science 333, 776–778. doi: 10.1126/science.1207745 21764755

[B71] Stebner F. Schuster C. Weber X. L. Greiff S. Leutner D. Wirth J. (2022). Transfer of metacognitive skills in self-regulated learning: effects on strategy application and content knowledge acquisition. Metacogn. Learn. 17, 715–744. doi: 10.1007/s11409-022-09322-x

[B72] Stewart M. (2021). “Understanding learning: theories and critique,” in University Teaching in Focus, eds. L. Hunt and D. Chalmers (London: Routledge), 3–28.

[B73] Sudarma I. K. Prabawa D. G. A. P. Suartama I. K. (2022). The application of information processing theory to design digital content in learning message design course. Int. J. Inf. Educ. Technol. 12, 1043–1049. doi: 10.18178/ijiet.2022.12.10.1718

[B74] Sui C. J. Yen M. H. Chang C. Y. (2024). Investigating effects of perceived technology-enhanced environment on self-regulated learning. Educ. Inf. Technol. 29, 161–183. doi: 10.1007/s10639-023-12270-x

[B75] Sun J. Wang Y. Hu Z. Liu Y. Sun Y. (2025). Cognitive offloading bias in primary and secondary school students and its relationship with metacognitive monitoring. Br. J. Dev. Psychol. 43, 645–659. doi: 10.1111/bjdp.12540 39676324

[B76] Turner C. (2022). Neuromedia, cognitive offloading, and intellectual perseverance. Synthese 200:66. doi: 10.1007/s11229-022-03472-w

[B77] Urbina S. Villatoro S. Salinas J. (2021). Self-regulated learning and technology-enhanced learning environments in higher education: a scoping review. Sustainability 13:7281. doi: 10.3390/su13137281

[B78] Wahn B. Schmitz L. Gerster F. N. Weiss M. (2023). Offloading under cognitive load: humans are willing to offload parts of an attentionally demanding task to an algorithm. PLoS ONE 18:e0286102. doi: 10.1371/journal.pone.0286102 37205658 PMC10198496

[B79] Warren L. Reilly D. Herdan A. Lin Y. (2021). Self-efficacy, performance and the role of blended learning. J. Appl. Res. High. Educ. 13, 98–111. doi: 10.1108/JARHE-08-2019-0210

[B80] Wei L. (2023). Artificial intelligence in language instruction: impact on English learning achievement, L2 motivation, and self-regulated learning. Front. Psychol. 14:1261955. doi: 10.3389/fpsyg.2023.1261955 38023040 PMC10658009

[B81] Weis P. P. Wiese E. (2022). Know your cognitive environment! Mental models as crucial determinant of offloading preferences. Hum. Factors 64, 499–513. doi: 10.1177/0018720820956861 32955351

[B82] Windasari A. Syefrinando B. Wiliyanti V. Komikesari H. Yuberti. (2024). “The influence of the blended learning model on students' concept understanding ability viewed from self-confidence,” in AIP Conference Proceedings, Vol. 3058 (New York: AIP Publishing LLC). p. 020013.

[B83] Wolf E. J. Harrington K. M. Clark S. L. Miller M. W. (2013). Sample size requirements for structural equation models. Educ. Psychol. Meas. 73, 913–934. doi: 10.1177/0013164413495237 PMC4334479 25705052

[B84] Xin X. Zhang M. (2024). Effects of flipped English learning designs on learning outcomes and cognitive load: workload of out-of-class activities versus during-class activities. J. Comput. Assist. Learn. 40, 1745–1765. doi: 10.1111/jcal.12978

[B85] Xu M. M. Tian Q. Yu S. H. Liu Y. T. Cao M. L. Zhang W. (2023). Cognitive engagement of nursing undergraduates in blended learning: a parallel mixed method study. Nurse Educ. Today 130:105947. doi: 10.1016/j.nedt.2023.105947 37660588

[B86] Yu Z. Xu W. Sukjairungwattana P. (2022). Meta-analyses of differences in blended and traditional learning outcomes and students' attitudes. Front. Psychol. 13:926947. doi: 10.3389/fpsyg.2022.926947 36186290 PMC9524290

[B87] Zimmerman B. J. (2000). “Attaining self-regulation: a social cognitive perspective,” in Handbook of Self-Regulation, eds. M. Boekaerts, P. R. Pintrich, and M. Zeidner (San Diego, CA: Academic Press), 13–39.

[B88] Zimmerman B. J. (2008). Investigating self-regulation and motivation: historical background, methodological developments, and future prospects. Am. Educ. Res. J. 45, 166–183. doi: 10.3102/0002831207312909

[B89] Zimmerman B. J. Schunk D. H. (2011). “Self-regulated learning and performance: an introduction and an overview,” in Handbook of Self-Regulation of Learning and Performance, eds. B. J. Zimmerman and D. H. Schunk (London: Routledge), 15–26.

[B90] Zou L. Zhang Z. Mavilidi M. Chen Y. Herold F. Ouwehand K. . (2025). The synergy of embodied cognition and cognitive load theory for optimized learning. Nat. Hum. Behav. 9, 877–885. doi: 10.1038/s41562-025-02152-2 40119235

